# Ghrelin: an anti-inflammatory theurapeutic agent in septic rats

**DOI:** 10.1186/cc14046

**Published:** 2014-12-03

**Authors:** GA Uluçay, E Özkök, H Yorulmaz, İ Aydın, Ş Tamer

**Affiliations:** 1 Department of Physiology, Faculty of Medicine, University of Istanbul, Turkey; 2 Institute of Experimental Medicine Research, Department of Neuroscience, University of Istanbul, Turkey; 3 Faculty of Health Science, University of Halic, Turkey; 4 Department of Histology, Faculty of Medicine, University of Istanbul, Istanbul, Turkey

## Introduction

Sepsis is a life-threatening systemic inflammatory syndrome (SIRS), which affects many organ systems, that leads to hemodynamic changes in the presence of suspected or proven infection, advancing to organ dysfunction and failure [1-3]. In recent studies, 377 out of 100,000 cases of sepsis were observed while $14,600,000,000 has been determined as the average annual hospital costs. Although there are many studies, the molecular mechanism is not yet clearly elucidated [2,3]. Lipopolysaccharide (LPS) is the lipid molecule which the outer membrane of Gram-negative bacteria used in designing the experimental sepsis model [4]. Ghrelin was discovered in 1999 as a specific ligand for growth hormone secretagogue receptor and then pleiotropic effects such as anti-inflammatory, antioxidant, and so forth were found. Ghrelin was released in many tissues and organs in which there also was its receptor. The liver is an organ which has ghrelin receptors and was affected by sepsis primary [5,6].

## Methods

In our study, male Wistar albino rats of average body mass 200 to 250 g were separated into four groups including: Control (*n *= 10), LPS (*E. coli *055:B5, 5 mg/kg, *n *= 10), ghrelin (10 nmol/kg i.v., *n *= 10), LPS + ghrelin LPS (5 mg/kg, ghrelin 10 nmol/kg i.v., *n *= 10). Rats were decapitated 24 hours after first injection. We aimed in this study to investigate effects of ghrelin in sepsis which is created by LPS with sepsis descriptive parameters such as body temperature and leukocyte count with proinflammatory cytokine TNFα and anti-inflammatory cytokines IL-10 by ELISA, and hematoxylin and eosin stain for observed morphological changes.

## Results

**Figure 1:**
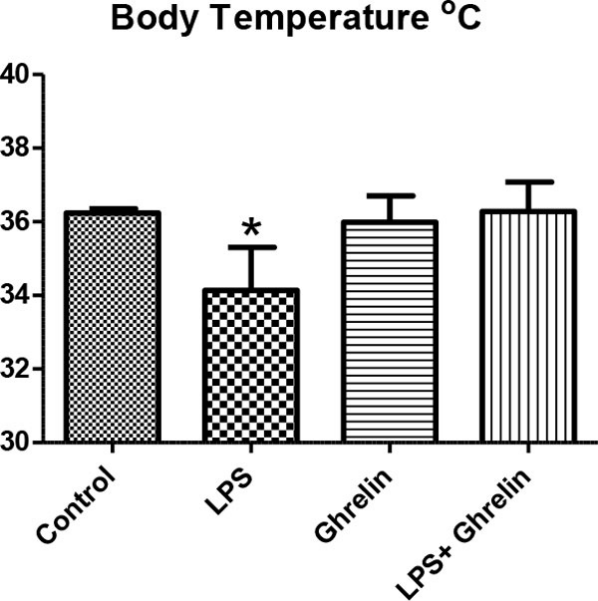


**Figure 2:**
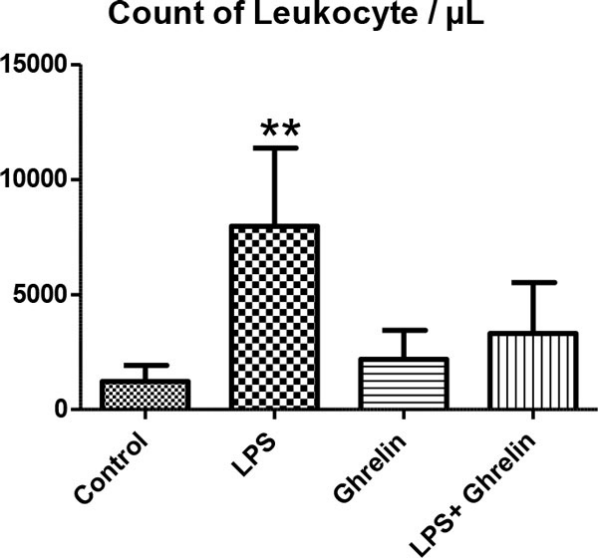


**Figure 3:**
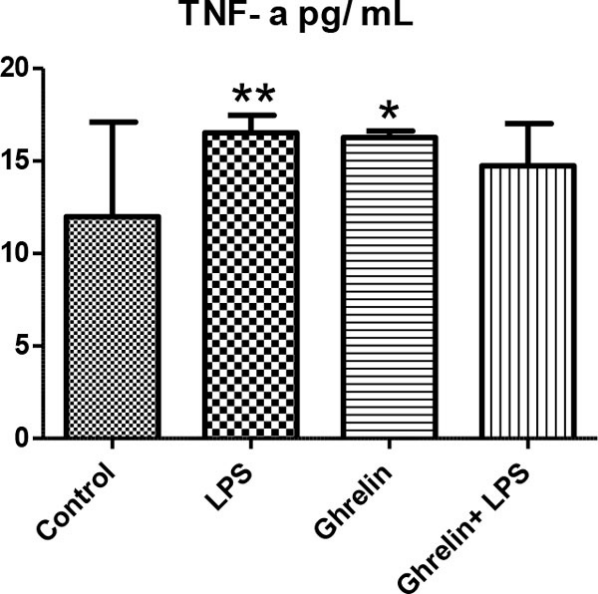


**Figure 4:**
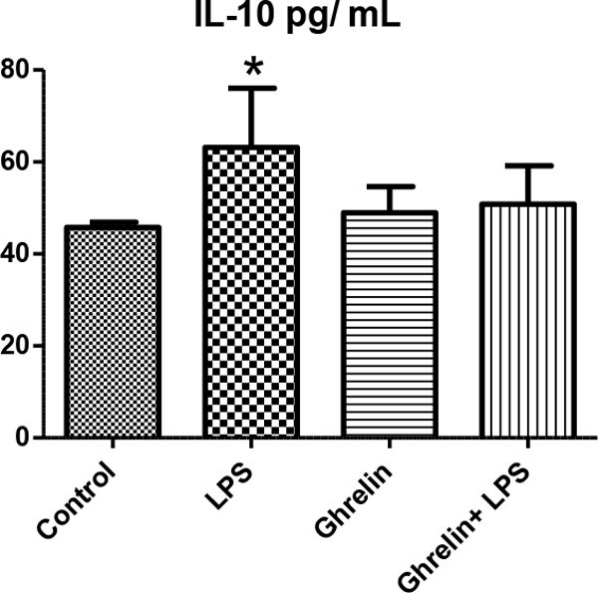


**Figure 5:**
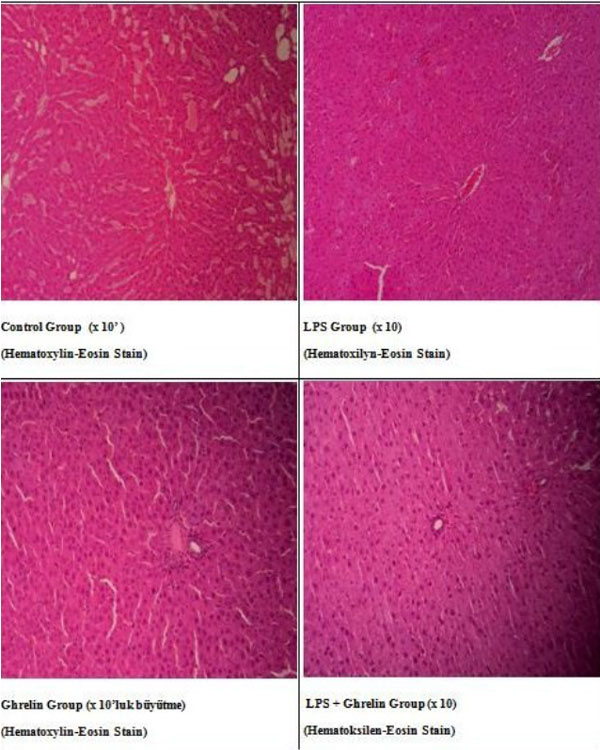


We detected the increase of leukocyte number, hypothermia, proinflammatory and anti-inflammatory cytokines such as TNFα and IL-10 as developing inflammatory reactions, resulting in hemodynamic and metabolic changes in rats treated with LPS. Also in groups with ghrelin treatment, ghrelin affects leukocyte numbers, body temperature, proinflammatory and anti-inflammatory cytokine levels and histological changes in controls and the LPS group (Figures 1 to 4). As a result of histologic examination, the curative effect of ghrelin partially on liver tissue damage is observed (Figure 5).

## Conclusion

We think the results of ghrelin may be affected depending on the dose and duration. Also partial healing effects of ghrelin in our results on this topic at the molecular level will contribute to other studies.
